# Novel Brominated Flame Retardants in Dust from E-Waste-Dismantling Workplace in Central China: Contamination Status and Human Exposure Assessment

**DOI:** 10.3390/toxics11010058

**Published:** 2023-01-06

**Authors:** Xuelin Li, Yu Wang, Wenbin Bai, Qiuyue Zhang, Leicheng Zhao, Zhipeng Cheng, Hongkai Zhu, Hongwen Sun

**Affiliations:** MOE Key Laboratory of Pollution Processes and Environmental Criteria, College of Environmental Science and Engineering, Nankai University, Tianjin 300350, China

**Keywords:** NBFRs, HBCDs, e-waste-dismantling area, dust ingestion, estimated daily intakes

## Abstract

Novel brominated flame retardants (NBFRs) have been widely used as alternatives to legacy BFRs. However, information on the contamination status and human exposure risks of electronic waste (e-waste)-derived NBFRs in the e-waste workplace is limited. In this study, six NBFRs and the legacy BFRs, hexabromocyclododecanes (HBCDs), were analyzed in 50 dust samples from an e-waste-dismantling workplace in Central China. The dust concentration of NBFRs in e-waste-dismantling workshops (median, 157–169 ng/g) was found to be significantly higher than those in an outdoor environment (17.3 ng/g) (*p* < 0.01). Differently, the highest median concentration of HBCDs was found in dust from the dismantling workshop for cellphones and computers (367 ng/g) among studied areas. The bis(2-ethylhexyl)-3,4,5,6-tetrabromo-phthalate (BEHTBP) was the predominant compound, which contributed 66.0–88.0% of measured NBFR concentrations. NBFRs might originate from plastic and rubber materials in wastes based on the correlation and principal component analysis. Moreover, the total estimated daily intakes (average scenario) of NBFRs were calculated at 2.64 × 10^−2^ ng/kg bw/d and 2.91× 10^−2^ ng/kg bw/d for the male and female dismantling workers, respectively, via dust ingestion, inhalation, and dermal contact pathways, which were lower than the reference dose values, and thus indicated a limited human exposure risk for NBFRs at the current level. Although the dust concentrations and daily intakes of NBFRs were still lower than those of other emerging pollutants (e.g., organophosphate and nitrogenous flame retardants) measured in the same sampling set, the elevated levels of NBFRs suggested the progressive BFR replacement process in China, which deserves more attention regarding their adverse effects on both the environment and human health.

## 1. Introduction

Brominated flame retardants (BFRs) such as polybrominated diphenyl ethers (PBDEs) and hexabromocyclododecane (HBCDs) are widely used in a variety of daily products including plastics, textiles, furniture, building materials, and electronics to reduce fire risk by interfering with the burning process of polymers [1,2]. BFRs dominated the organic flame-retardant market in the past 30 years [3]. However, since the toxic properties (persistence, bio-accumulation, etc.) of BFRs have been well-documented [4], penta-BDEs and octa-BDEs were regarded as persistent organic pollutants and were listed in the Stockholm Convention in 2009 [5]. Further, HBCDs and the deca-BDE were added to the Stockholm Convention in 2013 and 2017 [6,7], respectively.

The global restriction or phasing out of legacy BFRs causes the increasing market demand for flame retardants, thus inevitably leading to an increase in the use of their alternatives. Novel brominated flame retardants (NBFRs) have gradually become BFR substitutes with increasing global production (100,000 to 180,000 tons/year, 2019) [8,9]. 2,3,4,5,6-pentabromotoluene (PBT), hexabromobenzene (HBBZ), pentabromoethylbenzene (PBEB), 1,2-bis(2,4,6-tribromophenoxy)ethane (BTBPE), 2-ethylhexyl-2,3,4,5-tetrabromobenzoate (EHTBB), and bis(2-ethylhexyl)-3,4,5,6-tetrabromo-phthalate (BEHTBP) are the representative NBFRs with similar usages to legacy BFRs in a wide range of products [8,10,11]. The physico-chemical properties of NBFRs vary significantly (log *K*_ow_ 6.07–13.0, log *K*_oa_ 9.12–16.9, EPI suite estimation), which indicates the complexity of their environmental behaviors and risks. Furthermore, NBFRs are the additives in materials without chemical bonding, which eventually result in the release of these new chemicals into the environment [12,13].

NBFRs have been detected in various environmental matrices including the atmosphere [14,15], indoor air [4,16], dust [17], water [18,19,20], soil [21], and sediment [22,23,24]. A variety of sources were identified for NBFRs such as house environments, industrial processes, and especially waste recycling activities. NBFRs have been regarded as the “regrettable substitution” of BFRs since their potential adverse impacts were observed on human health and the ecological environment [13,25], including endocrine disruption, reproductive toxicity, cardiovascular toxicity, etc. [26]. However, until now, the information on the production, application, as well as the occurrence of NBFRs is limited, which creates difficulties in their environmental and health risk assessment [4].

An electronic waste (e-waste)-dismantling area is the typical “point source” area for various flame retardants, including legacy BFRs and emerging contaminants of organophosphate and nitrogen flame retardants. The One Health concept was integrated in the risk assessment and management of e-waste and called for the monitoring of e-waste-derived contaminants in the environment, animals, and humans [27]. The occurrence of NBFRs was found in e-waste facilities in Canada with the median concentration of 5540 ng/g in dust [28]. Notably, a million tons of e-waste is exported to developing countries such as China [27]. Few previous studies have found the occurrence of NBFRs in southern China, which was deemed as the world center of e-waste dismantling [29,30]. For example, the levels of NBFRs were 0.581 to 73,100 ng/g in sediment collected from an e-waste-dismantling area in southern China [31]. However, besides in southern China, the e-waste-dismantling industry is growing readily in Central and northern China [32,33,34], where the occurrence and risks of e-waste-related NBFRs merits further study. Additionally, the available data regarding NBFRs and other emerging flame retardants derived from e-waste usually originated from different individual workplaces, which limited their comparability. Therefore, investigating these flame retardants in the same typical workplace would provide more information on the replacement progress and current contamination status of flame retardants. Moreover, the exposure pathways of legacy BFRs in e-waste recycling area were suggested to be dust ingestion, air inhalation, dermal uptake, and diet [8], whereas similar exposure pathways for NBFRs need to be further elucidated.

In this study, six NBFRs and the legacy BFR HBCDs were analyzed in dust samples collected from a typical e-waste-dismantling workplace in Central China to (1) reveal the current contamination status of NBFRs in e-waste-dismantling workplaces; (2) compare the levels of NBFRs to other emerging flame retardants in the same studied area; and (3) estimate the occupational exposure risks of NBFRs in e-waste-dismantling workplaces via dust pathways.

## 2. Materials and Methods

### 2.1. Target Chemicals

Six NBFRs, including PBT, HBBZ, PBEB, BTBPE, EHTBB, and BEHTBP, as well as α-HBCD, β-HBCD, and γ-HBCD were analyzed. All target chemicals and surrogate standards information are provided in Supplementary Materials Section S1 and Table S1. The analyzed data regarding organophosphate and nitrogenous flame retardants in the same sample set from our previous studies [33,34] were included in this study for comparison.

### 2.2. Studied Area and Sample Collection

The studied area and sample collection were described in a previous study [33]. Briefly, a total of 50 dust samples were collected from an e-waste-dismantling workplace in Central China in 2020 (details in Supplementary Materials Section S2). The studied area included two dismantling workshops, namely dismantling workshop 1 (DW1) related to dismantling activities for cell phones and computers, and dismantling workshop 2 (DW2) related to the dismantling of refrigerators and washing machines. Additionally, dust samples from two outdoor areas were collected, being workshop outdoor (WO), which is an outdoor environment where dismantling activities do not take place, and residential area outdoor (RAO), which is the outdoor environment of a worker’s dormitory (around 200 m far away from DW1 and 2).

### 2.3. Sample Preparation and Instrumental Analysis

The extraction of NBFRs and HBCDs from dust samples followed the protocol reported in previous studies [35,36,37]. Briefly, dust samples (0.1 g) were mixed with surrogate standards (25 ng each) and extracted via acetone/n-hexane (1:1, *v*/*v*, 10 mL) with oscillation (20 min) and ultrasonication (20 min). Then, the extract was centrifuged (3000 r/min, 10 min), and the supernatant was collected. The sample was extracted twice, and the supernatants were combined. The collected supernatant was then divided into two equal fractions. These two fractions were concentrated to near dryness with nitrogen and then were reconstituted with methanol (0.5 mL) and n-hexane (0.5 mL) for the analysis of HBCDs and NBFRs, respectively. The prepared samples were stored at −20 °C prior to instrumental analysis.

NBFRs was analyzed via gas chromatography–mass spectrometry (7890A–5975C, Agilent, Santa Clara, CA, USA) with positive chemical ionization under the selective ion monitoring mode. Separation was conducted on a DB-5HT column (15 m × 0.25 mm i.d., 0.25 µm film thickness, Agilent, Santa Clara, CA, USA). The temperature program was set as: 90 °C held for 1 min, then ramped up to 240 °C by 20 °C/min, increased to 270 °C by 5 °C/min, and increased to 340 °C by 20 °C/min, then held for 2 min. Helium and methane were the carrier gas and reagent gas, respectively.

The analysis of HBCDs was performed using liquid chromatography–tandem mass spectrometry (1260–6460 B, Agilent, Santa Clara, CA, USA) coupled with a Nucleodex^®^ β-PM column (4 × 200 mm i.d., 5 µm, MN, Wiesbaden, Germany). The gradient of mobile phase was kept as Milli-Q water (10 mM NH_4_Ac): acetonitrile = 1:4 (v:v) with a flow rate of 0.4 mL/min for 16 min. The instrument was used with negative electron spray ionization in multiple reaction monitoring mode for quantification. Details of the instrumental analysis of the target chemicals are presented in Table S2.

### 2.4. Quality Assurance and Quality Control (QA/QC)

The matrix-spiked recoveries of NBFRs and HBCDs (100 ng/g) were 86.5–106% and 97.1–102%, respectively. Trace levels of NBFRs and HBCDs were found in procedure blanks at 1.6–3.90 ng for HBCDs and 7.34–15.4 ng for NBFRs. Additionally, no background level was found in the traveling blank samples. For target compounds without a procedure blank detected, their method determination limits (MDLs) were calculated on the basis of the limits of quantitation of instrumental analysis, whereas for those with a procedure blank detected, the MDLs were calculated by three times of standard deviations of the procedure blank values. The MDLs for NBFRs and HBCDs were 0.02–5.14 ng/g and 0.18–2.88 ng/g, respectively. Details of QA/QC data are presented in Table S2.

### 2.5. Risk Assessment

The estimated daily intake (EDI) of NBFRs and HBCDs in an e-waste-dismantling area via dust exposure in three pathways of ingestion, inhalation, and dermal contact were calculated using the following equations [38,39,40]:

(1) $${\mathrm{EDI}}_{\mathrm{ingestion}}=\frac{C\times {\mathrm{CF}}_{1}\times {\mathrm{IR}}_{\mathrm{ingestion}}\times \mathrm{EF}}{\mathrm{BW}}$$

(2) $${\mathrm{EDI}}_{\mathrm{inhalation}}=\frac{C\times {\mathrm{CF}}_{2}\times {\mathrm{IR}}_{\mathrm{inhalation}}\times \mathrm{EF}}{\mathrm{PEF}\times \mathrm{BW}}$$

(3) $${\mathrm{EDI}}_{\mathrm{dermal}-\mathrm{contact}}=\frac{C\times {\mathrm{CF}}_{1}\times \mathrm{EF}\times \mathrm{ABS}\times \mathrm{SA}\times \mathrm{AF}}{\mathrm{BW}}$$

(4) $${\mathrm{EDI}}_{\mathrm{sum}}={\mathrm{EDI}}_{\mathrm{ingestion}}+{\mathrm{EDI}}_{\mathrm{inhalation}}+{\mathrm{EDI}}_{\mathrm{dermal}-\mathrm{contact}}$$

where EDI_ingestion_, EDI_inhalation_, and EDI_dermal-contact_ are the EDIs (ng/kg bw/d) through dust ingestion, inhalation, and dermal contact, respectively. *C* is the concentration of BFRs measured in dust (ng/g); CF is the conversion factor; IR is the intake rate (mg/d, m^3^/d); EF is the exposure frequency (min/d); BW is the body weight (kg); PEF is the particle emission factor (m^3^/kg); ABS is the absorption fraction; SA is the skin surface area (cm^2^); AF is the adherence factor of dust (mg/cm^2^/d). The assigned values of parameters are shown in Table S3. Two exposure scenarios, namely an average and high exposure scenario, were assessed based on the median and the 95th percentile concentration, respectively.

The health risks of NBFRs for workers via dust ingestion, inhalation, and dermal contact in dismantling workshops were assessed by hazard quotients (HQs), which were calculated as the ratio of EDI_sum_ to the reference dose (RfD). The RfD values of the studied compounds were obtained from the Integrated Risk Information System from the U.S. EPA (Table S3). When the HQs were higher than 1, the assessed chemicals were considered a risk to humans [35].

### 2.6. Statistical Analysis

In the statistical analysis, concentrations of NBFRs and HBCDs below the MDLs were assigned a value at 1/2 MDLs. If the detection frequencies of NBFRs and HBCDs were <50.0%, the data were excluded from the statistical analysis. The Spearman correlation analysis was carried out to analyze the correlations among the studied NBFRs and HBCDs. The Mann–Whitney U test was conducted to analyze the differences in the measured concentrations between the studied areas. A principal component analysis (PCA) was carried out to reveal the similarities of the distribution patterns of the studied NBFRs and to analyze their potential sources on the basis of their concentrations. A Spearman correlation analysis, Mann–Whitney U test, and PCA were carried out using SPSS software (Version 22.0, SPSS Inc., Armonk, NY, USA).

## 3. Results and Discussion

### 3.1. NBFRs in Dust

All of the studied NBFRs were detected in dust samples from DW1 with the detection frequencies (DFs) of 60.0–100% (Table 1). EHTBB, PBT, and BTBPE were found with high DFs (73.0–100%) in dust samples from both the workshop and outdoor environment. The NBFR compounds of HBBZ, BEHTBP, and PBEB showed decreasing DFs in dust from the workshop to the outdoor environment. Especially for HBBZ and PBEB, the DFs of these two NBFRs were 0% in RAO. The high DFs of PBT and BTBPE were consistent with those reported in the earlier studies on e-waste recycling stations and urban houses in southern China [29,41].

**Figure 1:**
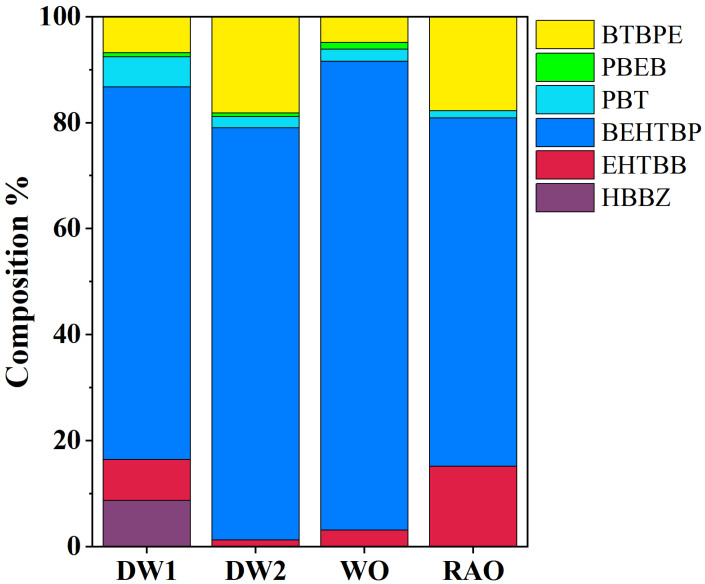
Composition profiles of NBFRs in dust samples from dismantling workshop 1 (DW1), dismantling workshop 2 (DW2), workshop outdoor (WO), and residential area outdoor (RAO).

The median concentrations of total NBFRs (Σ6NBFRs) in dust from DW1 was 157 ng/g with the range of 43.3–379 ng/g, which is comparable with that of Σ6NBFRs in dust from DW2 (median, range; 169, 70.2–1694 ng/g). The dust samples from the outdoor environment (WO and RAO) presented significant lower Σ6NBFR concentrations than those from the e-waste-dismantling workshop, especially for RAO, where only 17.3 ng/g of Σ6NBFR was detected in dust (*p* < 0.01). These results suggested the waste-dismantling activities might be an important source of NBFRs. BEHTBP was found as the predominating compound, with 96.5 ng/g in dust from DW1, among the measured NBFRs. Additionally, the concentrations of BEHTBP in dust from DW1 were much higher than those from RAO (9.72 ng/g). The wide application of BEHTBP in electronic and plastic products might cause the elevated levels of this chemical in e-waste-dismantling areas [42]. Furthermore, the NBFR compound of BTBPE exhibited the highest concentration in dust from DW2 among the studied areas, which suggested this chemical is related closely with products of washing machines and refrigerators. BTBPE was reported as the main additive in acrylonitrile–butadiene–styrene (ABS) and high-impact polystyrene (HIPS), thermoplastics, etc. [8]. ABS and HIPS can be used in washing machines, refrigerators, and other equipment shells, which may have been the reason for its high content in DW2 in our study [5,43,44,45,46]. In all the studied areas, BEHTBP was the predominant NBFR compound and was attributed to 66.0–88.0% of the measured NBFR concentrations. Similar composition profiles were found among the four studied areas (Figure 1), which might indicate the transfer potential of NBFRs from DW1 and DW2 to the outdoor environment.

**Figure 2:**
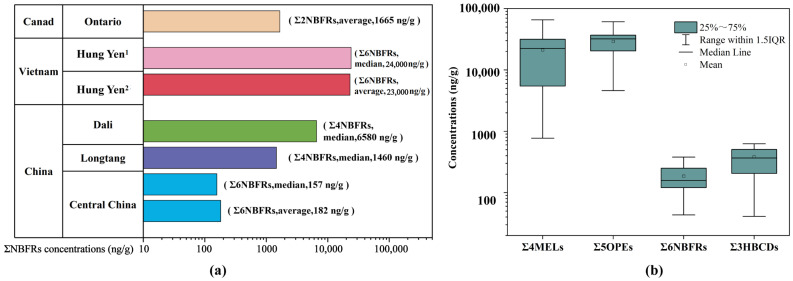
The dust concentrations of NBFRs in e-waste-dismantling workshop from different regions; Hung Yen^1^ is the data for samples collected in October 2015, Hung Yen^2^ is that in September 2019, Central China is the data from this study (**a**), and the dust concentrations of NBFRs and organophosphate and nitrogenous flame retardants in studied area; IQR means inter-quartile range (**b**).

The occurrence of NBFRs in dust samples from e-waste-dismantling areas reported in the literature is summarized in Figure 2a and Table S4. The median concentration of Σ6NBFRs in this study (157 ng/g) was found to be lower than that in dismantling workshops in Vietnam (Σ6NBFRs 24,000 ng/g) and southern China (Dali, Longtang, Σ4NBFRs 1460–6580 ng/g) (Figure 2a). The median concentration of BEHTBP in workshop dust from Central China found in this study (96.5 ng/g) was comparable with that in indoor dust from an e-waste area in southern China (49.0–193 ng/g) [29] (Table S4). However, the dust collected from an e-waste workplace in Canada (1940–2710 ng/g) showed much higher concentrations of BEHTBP than our results. Additionally, the concentrations of the typical NBFRs BEHTBP and EHTBB in workshop dust from European countries were 20 times higher than those found in dust samples from China. Therefore, the distribution of NBFRs showed regional differences in e-waste-dismantling areas [47]. Additionally, BEHTBP was the predominant NBFR compound in workshop dust from Central China and Canada [47], whereas HBBZ and BTBPE were the predominant compounds in Vietnam and southern China [29,48], which might indicate the difference in NBFR species used in these regions.

### 3.2. Comparison between NBFRs and Legacy BFRs

The median concentration of Σ3HBCDs in dust from DW1 was 367 ng/g, which was significantly higher than those concentrations found in dust samples collected from DW2 (110 ng/g), WO (60.8 ng/g), and RAO (5.16 ng/g) (*p* < 0.01). The decrease in Σ3HBCD concentrations in dust from DW1 to RAO suggested the emissions from e-waste represented the major source of legacy BFRs (Table 1). Interestingly, the concentration of Σ6NBFR in DW1 was much lower than that of Σ3HBCD, whereas in the other three studied areas, the concentrations of Σ6NBFRs were higher than those of Σ3HBCDs. This result might indicate the NBFRs and HBCDs had different sources which depended on the types of dismantled wastes. Additionally, although the dust concentrations of HBCDs in DW1 were still higher than those of NBFRs, they were already detected in the same order of magnitude, which suggested that the legacy BFRs are being progressively replaced by NBFRs in electronic products in China. Moreover, the concentrations of legacy BFRs of BDE-209 found in indoor dust from an e-waste recycling area in southern China (median, 55,100 ng/g) and Canada (96,700 ng/g) were higher than those of the NBFRs of EHTBB (60–1340 ng/g) and BEHTB (49–1990 ng/g) [29,47]. However, the Σ4NBFR concentrations (1460–50,010 ng/g) were found comparable to Σ8PBDE concentrations (644–55,100 ng/g) in an e-waste recycling area in southern China [29]. Therefore, the elevated concentrations of NBFRs in these “point source” areas raise the concern of exposure risks for waste-dismantling workers.

### 3.3. Correlations among NBFR Compounds

**Figure 3:**
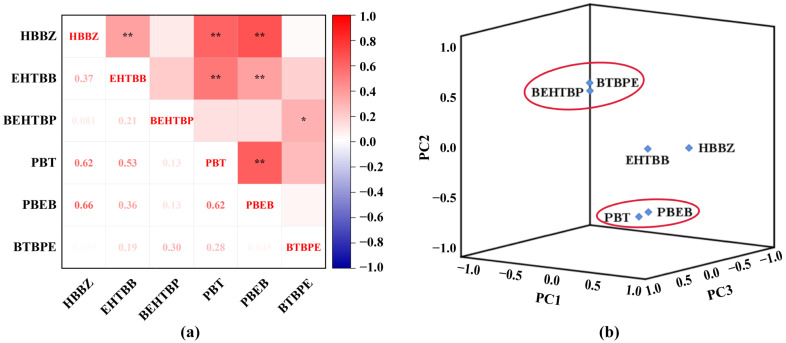
Spearman correlation coefficient among concentrations of NBFRs in dust samples from e-waste-dismantling workplace (* *p* < 0.05; ** *p* < 0.01) (**a**), and principal component analysis of NBFRs in dust samples from e-waste-dismantling workplace (PC1, 36.0% variance; PC2, 20.9% variance; PC3, 20.2% variance) (**b**).

The concentrations of PBT in dust samples were correlated significantly with those of EHTBB (Spearman correlation coefficient, R = 0.619, *p* < 0.01), BEHTBP (R = 0.528, *p* < 0.01), and PBEB (R = 0.624, *p* < 0.01) (Figure 3a), implying that these NBFR compounds have similar sources and applications. PBT is mainly used for plastic polymers (unsaturated polyester, polyethylene, polypropylene, etc.), textile, and rubber [49]. Similar applications of EHTBB, BEHTBP, and PBEB are also reported for rubber, plastics, and thermosetting polyester resins (textiles, wire and cable coatings, polyurethane foams, etc.), respectively [8,50,51]. Moreover, significant correlations were found between the concentrations of NBFRs (PBT and PBEB) and legacy BFRs (HBCDs) (R = 0.398–0.591, *p* < 0.01) (Figure 3a), which suggested their similar usage and emission sources related to electronic products. The penitential sources of the studied NBFRs were analyzed via PCA (Figure 3b). The NBFR compounds of PBT (0.675) and PBEB (0.590) showed similar loading on PC1 as HBCDs (0.657–0.734) (Table S5), which was consistent with the results of their significant Spearman correlations. HBCDs are typical BFRs related to electronic products [52]. Hence, the PC1 might be related to e-waste-releasing sources. BEHTBP showed similar loading to BTBPE on PC2 and PC3. BEHTBP is produced in 100–1000 tonnes per year and is widely used in plastic and rubber products [4]. Therefore, PC2 and PC3 might indicate the emission sources of plastic and rubber parts in e-waste dismantled in the studied areas.

### 3.4. Comparison between NBFRs and Other Emerging Flame Retardants

The survey data regarding flame retardants from the literature usually exhibited significant regional differences. Hence, a comparison between NBFRs and other emerging flame retardants in the same sampling set would have more practical significance than that carried out on the basis of the data from different areas. In our previous studies, the occurrence and distribution of organophosphate ester flame retardants (OPEs) and nitrogen flame retardants (melamine, MEL) were investigated in the same area as this study [33,34]. The median total concentrations of OPEs (34,900 ng/g) were comparable with those of MELs (22,365 ng/g), which were significantly higher than the total concentrations of NBFRs (Figure 2b). Specifically, the median concentration of a typical OPE compound of AO168 = O was 12,000 ng/g in dust from DW1, and that of the predominated MEL was 15,346 ng/g, which was much higher than the concentrations of the predominating NBFR BEHTBP. Therefore, NBFRs were not the dominant organic flame retardants in the studied e-waste workplace in terms of concentrations. However, toxicity studies regarding NBFRs as well as OPEs and MELs are limited, which may cause the high uncertainties of their risks.

### 3.5. Occupational Exposure Assessment

**Figure 4:**
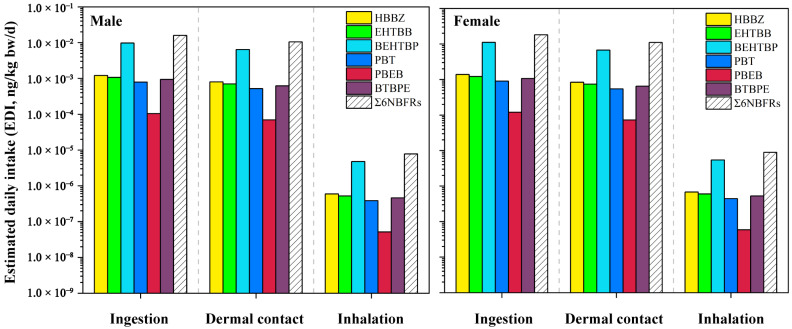
The occupational exposure of NBFRs in the e-waste-dismantling workshop (DW1, average exposure scenario) via dust pathways of ingestion, dermal contact, and inhalation.

The discussed EDI values in this section were all based on the average exposure scenario. The EDIs of Σ6NBFRs via dust ingestion, inhalation, and dermal contact in DW1 were 1.59 × 10^−2^ ng/kg bw/d, 7.76 × 10^−6^ ng/kg bw/d, and 1.05 × 10^−2^ ng/kg bw/d for male workers, and 1.82 × 10^−2^ ng/kg bw/d, 8.88 × 10^−6^ ng/kg bw/d, and 1.10 × 10^−2^ ng/kg bw/d for female workers under the average exposure scenario, respectively (Figure 4, Tables S6–S8). The mass-based intake rates of ingestion (IR_ingestion_), inhalation (IR_inhalation_/PEF), and dermal contact (ABS × SA × AF) were 20 mg/d, 9.79 × 10^−3^ mg/d, and 12.1–13.2 mg/d, respectively; thus, the calculated EDI value of dust ingestion was estimated to be higher than inhalation and dermal contact accordingly. Among the studied NBFRs, the exposure values of BEHTBP via dust ingestion (male, 3.32 × 10^−2^ ng/kg bw/d; female, 3.80 × 10^−2^ ng/kg bw/d) were estimated to be higher than those via inhalation (male, 1.62 × 10^−5^ ng/kg bw/d; female, 1.86 × 10^−5^ ng/kg bw/d) and dermal contact (male, 2.20 × 10^−2^ ng/kg bw/d; female, 2.30 × 10^−2^ ng/kg bw/d). Therefore, dust ingestion was the main exposure pathway of NBFRs among the three studied pathways.

No significant difference in the EDI_ingestion_ values of Σ6NBFR was found between DW1 (male, 1.59 × 10^−2^ ng/kg bw/d; female, 1.82 × 10^−2^ ng/kg bw/d) and DW2 (male, 1.64 × 10^−2^ ng/kg bw/d; female, 1.88 × 10^−2^ ng/kg bw/d), whereas the EDI_ingestion_ values estimated in the workshop were much higher than those in the residential area (male, 5.90 × 10^−4^ ng/kg bw/d; female, 4.60 × 10^−4^ ng/kg bw/d). Among different regions, the difference in EDI is driven by the measured concentrations according to the calculation model. The concentrations of NBFRs in DW1 and DW2 were higher than in the outdoor area, which resulted in the higher calculated EDIs in dismantling workshop areas. Additionally, the EDI_ingestion_ of BTBPE (male, 9.37 × 10^−4^ ng/kg bw/d; female, 1.07 × 10^−3^ ng/kg bw/d) and HBBZ (1.21 × 10^−3^ ng/kg bw/d, 1.39 × 10^−3^ ng/kg bw/d) for workers in DW1 (EF 0.33, equivalent to 8 working hours per day) was higher than that for Chinese adults in a house environment (BTBPE 2.20 × 10^−4^ ng/kg bw/d, HBBZ 4.84 × 10^−5^ ng/kg bw/d, EF 0.64) [53]. Therefore, the workers in the waste-dismantling workplace suffered from more NBFR exposure than ordinary people. Moreover, the EDI values of NBFRs via dust ingestion in this study reached the same order of magnitude as those of legacy BFR HBCDs (male, 3.70 × 10^−2^ ng/kg bw/d; female 4.23 × 10^−2^ ng/kg bw/d; DW1), which indicated the considerable exposure levels of these emerging BFRs.

The EDI_ingestion_ values of BEHTBP and EHTBB for workers estimated in this study were lower than those reported in a Canadian e-waste-dismantling facility (1.50 ng/kg bw/d and 0.34 ng/kg bw/d, EF 0.33) [47]. Moreover, the EDI_ingestion_ values of Σ4NBFRs among e-waste-dismantling areas in southern China (0.42–14.3 ng/kg bw/d, EF 0.33) [29] were 10 times higher than those estimated in this study. Hence, the exposure levels of NBFRs for e-waste-dismantling workers also exhibited regional differences. In the same studied e-waste area, the calculated EDI_ingestion_ values of Σ4MELs and Σ5OPEs for e-waste-dismantling workers were 9.70 ng/kg bw/d (EF 0.42) and 3.29 ng/kg bw/d (EF 0.33–0.43) [33,34], which were also higher than the EDI_ingestion_ values of Σ6NBFRs for workers in DW1. In this study, the calculated HQs of all target BFRs based on dust ingestion, inhalation, and dermal contact pathways were below 1 (Table S9), which suggested that the exposure to NBFRs via dust in waste-dismantling workplaces might pose a limited health risk for workers under the current exposure levels.

In the two studied dismantling workshops, the workers wear gloves and masks when carrying out dismantling activities, which may partially prevent direct contact with dust. Therefore, the calculated EDI values in this study may have been overestimated compared to the actual situation. More accurate parameters of dust intake rate which consider the protection measures should be investigated and applied in future exposure studies regarding e-waste-dismantling workers. Additionally, since dust ingestion was suggested as the major exposure pathway of NBFRs, wearing gloves and masks during the working time and washing hands after dismantling activities would be efficient ways to minimize the occupational exposure to NBFRs in e-waste-dismantling workshops.

## 4. Conclusions

In this study, the distribution of NBFRs and HBCDs in indoor and outdoor dust samples from e-waste-dismantling areas in Central China as well as their human exposure levels were investigated. The e-waste-dismantling workshop showed higher concentrations of NBFRs and HBCDs than the outdoor environment did. The BEHTBP was found as the predominant NBFR compound in the e-waste workshop. The results of the Spearman correlation analysis and PCA suggested similar sources of PBT and PBEB and BEHTBP and BTBPE. Moreover, the exposure assessment showed that the exposure levels of NBFRs via dust ingestion might be higher than inhalation and dermal contact, and the exposure levels of NBFRs were in the same order of magnitude as those of legacy BFR HBCDs under the average exposure scenario. Our results also revealed the dust concentrations of NBFRs were lower than those of OPEs and nitrogenous flame retardants in the same studied area. Although the concentration and exposure level of NBFRs are still lower than those of other emerging flame retardants, the adverse effects of NBFRs for both the environment and human health are noteworthy due to the limited knowledge of their toxicities.

## Figures and Tables

**Table 1:** Concentrations (ng/g) and detection frequencies (DF, %) of NBFRs and HBCDs in dust samples from e-waste-dismantling workplace.

Target Compounds		HBBZ	EHTBB	BEHTBP	PBT	PBEB	BTBPE	Σ6NBFRs	Σ3HBCDs *
DW1 (*n* = 20)	Median	12.0	10.6	96.5	7.83	1.04	9.30	157	367
	Average	22.3	11.5	126	9.35	1.00	16.1	182	383
	Range	n.d.-101	2.02–40.0	n.d.-332	3.91–25.5	0.62–1.65	<MDL-59.1	43.3–379	367–1155
	DF	60%	100%	85%	100%	100%	95%		
DW2 (*n* = 10)	Median	n.d.	1.78	111	3.09	1.00	25.9	169	110
	Average	281	4.20	158	10.2	1.76	41.9	497	789
	Range	n.d.-1173	<MDL-15.3	32.1–420	0.40–35.3	n.d.-4.79	7.40–145	70.2–1694	8.54–4542
	DF	30%	80%	100%	100%	80%	100%		
WO (*n* = 15)	Median	n.d.	2.61	74.6	1.95	1.07	4.11	130	60.8
	Average	83.1	4.08	126	22.5	15.8	4.56	179	372
	Range	n.d.-923	<MDL-18.7	32.4–337	0.87–299	<MDL-222	<MDL-9.48	39.5–1690	11.5–602
	DF	27%	87%	100%	100%	60%	73%		
RAO (*n* = 5)	Median	n.d.	2.24	9.72	0.21	n.d.	2.62	17.3	5.16
	Average	n.d.	3.76	323	0.90	n.d.	50.0	377	110
	Range	n.d.-n.d.	0.85–11.4	<MDL-1546	0.05–2.10	n.d.-n.d.	<MDL-241	7.09–1799	2.94–498
	DF	0%	100%	60%	100%	0%	80%		

DW1: dismantling workshop 1; DW2: dismantling workshop 2; WO: workshop outdoor; RAO: residential area outdoor. MDL: method detection limit; n.d. = non-detected. * Total concentrations of α-HBCD, β-HBCD, and γ-HBCD.

## Data Availability

Data available in a publicly accessible repository. The data presented in this study are openly available in https://doi.org/10.3390/xxxxx.

## Supplementary Materials

The following supporting information can be downloaded at: https://www.mdpi.com/article/10.3390/toxics11010058/s1, Table S1. Chemical properties of target NBFRs and HBCDs compounds; Table S2. Instrument performance, method detection limits (MDLs), and matrix spike recoveries of HBCDs and NBFRs analysis; Table S3. The parameters for the estimated daily intake and hazard quotient calculation; Table S4. The occurrence of NBFRs in dust samples from e-waste dismantling area and residential environment; Table S5. Component matrix of principal component analysis; Table S6. The estimated daily intake of NBFRs and HBCDs via dust ingestion pathway; Table S7. The estimated daily intake of NBFRs and HBCDs via dust inhalation pathway; Table S8. The estimated daily intake of NBFRs and HBCDs via dust dermal contact pathway; Table S9. The calculated hazard quotients for NBFRs and HBCDs [29,31,33,34,39,47,48,53,54,55,56,57,58,59,60,61].
