# Supplementary material for: Novel Brominated Flame Retardants in Dust from E-Waste-Dismantling Workplace in Central China: Contamination Status and Human Exposure Assessment
Source: Toxics. 2023 Jan 6;11(1):58. doi: 10.3390/toxics11010058 (PMC9864280; doi:10.3390/toxics11010058)
Supplement: Supplementary file 1 [file toxics-11-00058-s001.zip › toxics-2080215-supplementary.pdf]

Supplementary Information

Novel Brominated Flame Retardants in Dust from E-Waste-Dismantling Workplace in Central China: Contamination Status and Human Exposure Assessment

Xuelin Li, Yu Wang *, Wenbin Bai, Qiuyue Zhang, Leicheng Zhao, Zhipeng Cheng, Hongkai Zhu and Hongwen Sun

MOE Key Laboratory of Pollution Processes and Environmental Criteria, College of Environmental Science and Engineering, Nankai University, Tianjin 300350, China

* Correspondence: yu.wang@nankai.edu.cn

Number of pages: 14

Number of tables: 9

Contents

Section S1. Chemicals 3

Section S2. Sample collection........ 3

Table S1. Chemical properties of target NBFRs and HBCDs compounds........ 4

Table S2. Instrument performance, method detection limits (MDLs), and matrix spike recoveries of HBCDs and NBFRs analysis. 6

Table S3. The parameters for the estimated daily intake and hazard quotient calculation........ 7

Table S4. The occurrence of NBFRs in dust samples from e-waste dismantling area and residential environment........ 8

Table S5. Component matrix of principal component analysis........ 10

Table S6. The estimated daily intake of NBFRs and HBCDs via dust ingestion pathway (ng/kg bw/d)........ 11

Table S7. The estimated daily intake of NBFRs and HBCDs via dust inhalation pathway (ng/kg bw/d)........ 12

Table S8. The estimated daily intake of NBFRs and HBCDs via dust dermal contact pathway (ng/kg bw/d)........ 13

Table S9. The calculated hazard quotients for NBFRs and HBCDs 14

Section S1. Chemicals

Six NBFRs including 2,3,4,5,6-pentabromotoluene (PBT), hexabromobenzene (HBBZ), pentabromoethylbenzene (PBEB), 1,2-bis(2,4,6-tribromophenoxy)ethane (BTBPE), 2-ethylhexyl 2,3,4,5-tetrabromobenzoate (EHTBB), bis(2-ethylhexyl)-3,4,5,6-tetrabromo-phthalate (BEHTBP), as well as hexabromocyclododecane (α-HBCD, β-HBCD, γ-HBCD) was analyzed. The surrogate standards of $^{13}C_6$-HBBZ, d_{34}-BEHTBP, and d_{18}-HBCDs were used. The data obtained from previous studies (Zhang et al., 2021 , Zhao et al., 2022) on organophosphate flame retardants including tris (2-chloroethyl) phosphate (TCEP), triphenyl phosphate (TPHP), tris(2,4-di-tert-butylphenyl) phosphate (AO168=O), bis(2,4-di-tert-butylphenyl) pentaerythritol diphosphate (AO626=O_2), trisnonylphenol phosphate (TNPP) and nitrogenous flame retardants including melamine (MEL) and its derivatives melamine (CYA), aminoyl (AMD) and ammeline (AMN) were also included in this study.

Section S2. Sample collection

Totally 50 dust samples were collected from an e-waste dismantling area (280,000 m2, dismantling amount > 100,000 sets/day) in Central China from October to November 2020. The studied area included four sampling sections, namely dismantling workshop 1 (DW1) for phones, computers, and televisions, dismantling workshop 2 (DW2) for washing machines and refrigerators, workshop outdoor (WO), and residential area outdoor (RAO). The dust samples were collected by soft-bristled brush and vacuum cleaner (Deerma, DX901, 12 Kpa) with 25 μm pore size nylon sock to prevent cross-contamination. All the sampling tools are cleaned by methanol twice before use. After collection, all dust samples were wrapped in aluminum foil, sealed in individual bags, sent to the laboratory in 24 h, sieved by a pre-cleaned 100 μm mesh sieve, and stored at -20℃ for further analysis.

Table S1. Chemical properties of target NBFRs and HBCDs compounds.

Chemicals	Abbr.	CAS#	M.W.	Formula	Chemical structure	Solubility (mg/L) 25 ˚C	Vapor pressure at 25°C (mmHg)	Log K_{ow}	log K_{oc}	log K_{oa}	Half-lfe time (h) (Level III Fugacity Model; Air/Water/Soil/Sediment)	Daphnia magna LC_{50} (48hr) -Log10 (mol/L)
	α-HBCD											
1,2,5,6,9,10-Hexabromocyclododecane	β-HBCD	3194-55-6	641.70	$C_{12}H_{18}Br_6$	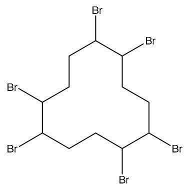	8.60E-03	5.25E-07	7.74	6.717	10.466	51.2/1.44E3/2.88E3/1.3E4	2.45
	γ-HBCD											
1,2-Bis(2,4,6-tribromophenoxy)ethane	BTBPE	37853-59-1	687.64	$C_{14}H_8Br_6O_2$	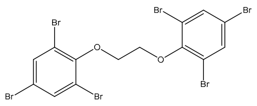	0.20	3.49E-09	9.15	6.098	15.674	17.3/4.32E3/8.64E3/3.89E4	/
2-Ethylhexyl-2,3,4,5-tetrabromobenzoate	EHTBB	183658-27-7	549.92	$C_{15}H_{18}Br_4O_2$	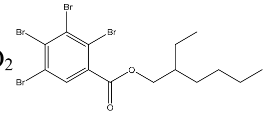	1.11E-02	1.13E-07	8.75	5.699	12.335	23.5/1.44E3/2.88E3/1.3E4	3.38
Bis(2-ethylhexyl)-3,4,5,6-tetrabromo-phthalate	BEHTBP	26040-51-7	706.14	$C_{24}H_{34}Br_4O_4$	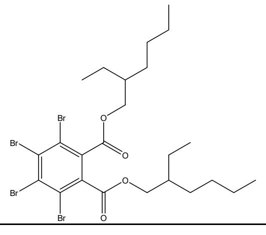	9.19E-03	1.50E-08	11.95	7.404	16.864	11.8/1.44E3/2.88E3/1.3E4	3.06

Chemicals	Abbr.	CAS#	M.W.	Formula	Chemical structure	Solubility (mg/L) 25 ˚C	Vapor pressure at 25°C (mmHg)	Log K_{ow}	log K_{oc}	log K_{oa}	Half-lfe time (h) (Level III Fugacity Model; Air/Water/Soil/Sediment)	Daphnia magna LC_{50} (48hr) -Log10 (mol/L)
Pentabromoethylbenzene	PBEB	85-22-3	500.65	$C_8H_5Br_5$	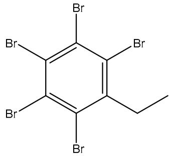	2.94E-03	2.97E-07	7.84	6.491	9.970	223/4.32E3/8.64E3/3.89E4	4.17
2,3,4,5,6-Pentabromotoluene	PBT	87-83-2	486.62	$C_7H_3Br_5$	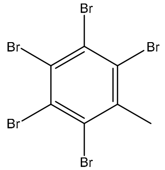	1.67E-02	5.68E-06	6.99	6.066	9.602	1.39E3/4.32E3/8.64E3/3.89E4	/
Hexabromobenzene	HBBZ	87-82-1	551.49	C_6Br_6	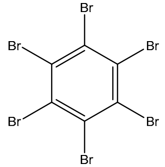	1.60E-04	1.38E-06	6.07	5.268	9.126	2.24E4/4.32E3/8.64E3/3.89E4	3.52

All the predicted data are generated using the US Environmental Protection Agency's EPI Suite™ (https://www.epa.gov/tsca-screening-tools); Kow (Octanol-Water Partition Coefficient, KOWWIN v1.69); Koc (Soil Adsorption Coefficient, KOCWIN v2.00); Koa (Octanol-Air Partition Coefficient, KOAWIN v1.10); Vapor pressure (MPBPVP v1.43, Modified Grain method); Water solubility (WSKOW v1.42); LC50 were assessed by T.E.S.T tool (Toxicity Estimation Software Tool,https://www.epa.gov/chemical-research/toxicity-estimation-software-tool-test)

Table S2. Instrument performance, method detection limits (MDLs), and matrix spike recoveries of HBCDs and NBFRs analysis.

Target Compounds	Quantitative ion	Retention time (min)	Surrogate standards	Linearity range (ng/g) (R2)	Procedure blank (ng)	Matrix spiked Recovery % (SD %) (100 ng/g)	MDL (ng/g)
HBBZ	551.5	6.76	13C6-HBBZ	1-450 (0.9984)	0.00	86.46 (7.6)	2.90
EHTBB	356.7	8.02	13C6-HBBZ	1-450 (0.9760)	15.4	97.85 (15.7)	0.74
BEHTBP	383.7	11.26	d34-BEHTBP	1-450 (0.9987)	7.34	106.23 (6.1)	5.14
PBT	81	6.02	13C6-HBBZ	1-450 (0.9893)	7.72	103.41(6.0)	0.02
PBEB	81	6.23	13C6-HBBZ	1-450 (0.9876)	8.39	103.53 (5.2)	0.53
BTBPE	81	11.16	d34-BEHTBP	1-450 (0.9868)	11.1	92.97 (10.7)	2.05
(-)-α-HBCD	640.6→80.9	5.95	(−)d18-α-HBCD	1.5-200 (0.9993)	3.22	97.91 (1.7)	0.26
(-)-β-HBCD	640.6→80.9	6.36	(−)d18-β-HBCD	1.5-200 (0.9976)	3.89	101.41 (1.9)	0.87
(+)-α-HBCD	640.6→80.9	6.77	(+)d18-α-HBCD	1.5-200 (0.9972)	3.94	98.25 (4.2)	0.18
(+)-β-HBCD	640.6→80.9	7.18	(+)d18-β-HBCD	1.5-200 (0.9988)	2.84	101.64 (2.7)	0.26
(+)-γ-HBCD	640.6→80.9	7.67	(+)d18-γ-HBCD	1.5-200 (0.9995)	3.05	101.98 (4.9)	0.36
(-)-γ-HBCD	640.6→80.9	9.97	(−)d18-γ-HBCD	1.5-200 (0.9994)	1.66	102.18 (1.2)	2.88

Table S3. The parameters for the estimated daily intake and hazard quotient calculation.

Parameters	Abbr.	Unit	Values		Reference
			Male	Female	
Ingestion rates	$IR_{ingestion}$	mg d^{-1}	20	20	(USEPA, 2011)
Inhalation rates	$IR_{inhalation}$	m^3 d^{-1}	13.3	13.3	(USEPA, 2011)
Exposure frequency	EF	min d^{-1}	480* (workshop)<br>163 (outdoor)	480* (workshop)<br>111 (outdoor)	(CRAES, 2015)
Absorbed fraction	ABS	unitless	0.03	0.03	(USEPA, 2011)
Skin surface area	SA	cm^2	4600	4200	(CRAES, 2015)
Adherence factor	AF	mg cm^{-2} d^{-1}	0.096	0.096	(USEPA, 2011)
Particle emission factor	PEF	m^3 kg^{-1}	1.36×10^9	1.36×10^9	(USEPA, 2011)
Body weight	BW	kg	66.1	57.8	(CRAES, 2015)
Conversion factor 1	CF_1		0.001	0.001	(CRAES, 2015)
Conversion factor 2	CF_2		1000	1000	(CRAES, 2015)
**Reference dose**					
HBBZ	Rfd	ng/kg bw/d	100		Integrated Risk Information System
EHTBB			20000		
BEHTBP			20000		
PBT			100		
PBEB			100		
BTBPE			243000		
α-HBCD			200000		
β-HBCD			200000		
γ-HBCD			200000		

*The EF of worker in workshop was set as 480 min/day (8 working hours per day) for both male and female workers.

RfD values are all from Integrated Risk Information System (IRIS) in EPA (https://www.epa.gov/ncea/iris/index.html).

Table S4. The occurrence of NBFRs in dust samples from e-waste dismantling area and residential environment

Area	Sampling time	Matrix (units)	Median concentration						Reference
			HBBZ	EHTBB	BEHTBP	PBT	PBEB	BTBPE	
Central China	2020	E-waste workshop dust (ng/g)	12.0	10.6	96.5	7.83	1.04	9.30	This study
Guiyu, South China	2020	E-waste sediment (ng/g, mean)	4.74	4.74	-	4.74	0.362	830	(Ling et al., 2022)
Taizhou, East China	2018	E-waste Water (ng/L, mean)	0.34	n.d.	-	0.28	0.012	0.030	(Ling et al., 2021)
		E-waste sediment (ng/g, mean)	23.0	1.60	-	1.30	0.02	32.0	
Hanoi Vietnam	2018	Urban house dust (ng/g)	-	-	-	-	0.25	1.10	(Hoang et al., 2021)
Ontario, Canada	2017	E-waste workshop floor dust	-	693	1940	-	-	-	(Nguyen et al., 2019)
		E-waste workshop workbench dust	-	738	2710	-	-	-	
Dalian, North China	2016-2017	$PM_{2.5}$ (pg/m^3)	4.43	0.20	0.66	0.20	0.06	0.10	(Wang et al., 2020)
		Gas (pg/m^3)	1.27	0.19	1.22	1.07	0.34	0.07	
Belgium	2016-2017	Indoor house dust (ng/g)	-	-	-	-	0.02	0.87	
Spain	2016-2017	Indoor house dust (ng/g)	-	-	-	-	0.03	1.58	(Torre et al., 2020)
Italy	2016-2017	Indoor house dust (ng/g)	-	-	-	-	0.06	1.67	
		Indoor floor dust	-	4.10	58.0	-	-	1.30	
Bui Dau, Vietnam	2015	E-waste workshop dust (ng/g, average)	1500	-	-	-	-	1300	(Wannomai et al., 2020)
Longtang, South China	2013	E-waste recycling sites indoor dust (ng/g)	-	7.50	88.0	-	-	28.0	(Zheng et al., 2015)
Dali,	2013	E-waste recycling sites	-	36.0	193	-	-	40.0	

Area	Sampling time	Matrix (units)	Median concentration						Reference
			HBBZ	EHTBB	BEHTBP	PBT	PBEB	BTBPE	
South China		indoor dust (ng/g)							
Guiyu, South China	2013	E-waste recycling sites indoor dust (ng/g)	-	60.0	49.0	-	-	3870	
Beijing	2012-2013	Indoor Elevated surface dust (ng/g)	-	3.50	30.0	-	-	1.70	(Bu et al., 2019)
South China	2007	E-waste house dust (ng/g)	21.0	-	-	0.79	1.83	20.0	(Wang et al., 2010)
	2008-2009	Urban house dust (ng/g)	18.1	-	-	1.52	0.15	6.47	
Belgian	2008	Homes dust (ng/g)	-	1.00	-	-	-	2.00	(Ali et al., 2011)
UK	2008	Schools dust (ng/g)	-	25.0	-	-	-	9.00	

Notes: n.d. means not detected; the dash means the compound is not measured

Table S5. Component matrix of principal component analysis.

	Component		
	1	2	3
HBBZ	0.881	0.032	0.032
EHTBB	0.460	-0.004	0.138
BEHTBP	0.144	0.627	0.682
PBT	0.675	-0.577	0.434
PBEB	0.590	-0.626	0.478
BTBPE	0.105	0.695	0.632
α-HBCD	0.734	0.146	-0.419
β-HBCD	0.657	0.266	-0.518
γ-HBCD	0.670	0.429	-0.275
Eigenvalues*	3.244	1.878	1.821
Variances (%)	36.04	20.87	20.24

* Eigenvalues ⩾ 1.0 were used to construct the PCA model.

Table S6. The estimated daily intake of NBFRs and HBCDs via dust ingestion pathway (ng/kg bw/d).

Areas	DW1				DW2				WO				RAO			
Gender	Male		Female		Male		Female		Male		Female		Male		Female	
EDIs	Average	High	Average	High	Average	High	Average	High	Average	High	Average	High	Average	High	Average	High
HBBZ	1.21E-03	8.36E-03	1.39E-03	9.56E-03	-*	1.14E-01	-	1.30E-01	-	1.31E-02	-	1.02E-02	-	-	-	-
EHTBB	1.07E-03	1.88E-03	1.22E-03	2.15E-03	1.80E-04	1.34E-03	2.06E-04	1.54E-03	8.91E-05	3.94E-04	6.94E-05	3.07E-04	7.67E-05	3.32E-04	5.97E-05	2.59E-04
BEHTBP	9.73E-03	3.32E-02	1.11E-02	3.80E-02	1.12E-02	4.11E-02	1.28E-02	4.70E-02	2.55E-03	9.72E-03	1.99E-03	7.57E-03	3.32E-04	4.27E-02	2.59E-04	3.32E-02
PBT	7.89E-04	1.83E-03	9.02E-04	2.09E-03	3.12E-04	3.16E-03	3.56E-04	3.62E-03	6.68E-05	3.26E-03	5.21E-05	2.54E-03	7.16E-06	7.17E-05	5.58E-06	5.59E-05
PBEB	1.05E-04	1.40E-04	1.20E-04	1.60E-04	1.01E-04	4.71E-04	1.15E-04	5.39E-04	3.64E-05	2.38E-03	2.84E-05	1.86E-03	-	-	-	-
BTBPE	9.37E-04	4.81E-03	1.07E-03	5.50E-03	2.61E-03	1.10E-02	2.98E-03	1.26E-02	1.41E-04	3.13E-04	1.10E-04	2.44E-04	8.97E-05	6.61E-03	6.99E-05	5.15E-03
∑6NBFRs	1.59E-02	3.73E-02	1.82E-02	4.26E-02	1.64E-02	1.54E-01	1.88E-02	1.76E-01	4.45E-03	2.56E-02	3.47E-03	2.00E-02	5.90E-04	4.96E-02	4.60E-04	3.86E-02
α-HBCD	6.92E-03	3.22E-02	7.91E-03	3.68E-02	5.35E-03	4.17E-02	6.12E-03	4.77E-02	8.56E-04	9.52E-03	6.67E-04	7.42E-03	9.78E-05	8.96E-03	7.62E-05	6.98E-03
β-HBCD	2.40E-03	7.14E-03	2.75E-03	8.17E-03	7.59E-04	1.13E-02	8.68E-04	1.30E-02	2.92E-04	1.64E-03	2.28E-04	1.28E-03	1.93E-05	4.57E-03	1.51E-05	3.56E-03
γ-HBCD	2.16E-02	5.41E-02	2.46E-02	6.19E-02	4.14E-03	2.74E-01	4.74E-03	3.13E-01	7.44E-04	4.56E-03	5.80E-04	3.55E-03	9.85E-05	9.86E-04	7.67E-05	7.69E-04
∑3HBCDs	3.70E-02	6.57E-02	4.23E-02	7.51E-02	1.12E-02	3.24E-01	1.29E-02	3.70E-01	2.08E-03	1.25E-02	1.62E-03	9.76E-03	1.76E-04	1.39E-02	1.38E-04	1.09E-02

*the dash means the median concentration is not available.

Table S7. The estimated daily intake of NBFRs and HBCDs via dust inhalation pathway (ng/kg bw/d).

Areas	DW1				DW2				WO				RAO			
Gender	Male		Female		Male		Female		Male		Female		Male		Female	
EDIs	Average	High	Average	High	Average	High	Average	High	Average	High	Average	High	Average	High	Average	High
HBBZ	5.93E-07	4.09E-06	6.78E-07	4.68E-06	-	5.56E-05	-	6.35E-05	-	6.42E-06	-	5.01E-06	-	-	-	-
EHTBB	5.22E-07	9.21E-07	5.96E-07	1.05E-06	8.79E-08	6.58E-07	1.01E-07	7.52E-07	4.36E-08	1.92E-07	3.39E-08	1.50E-07	3.75E-08	1.63E-07	2.92E-08	1.27E-07
BEHTBP	4.76E-06	1.62E-05	5.44E-06	1.86E-05	5.48E-06	2.01E-05	6.26E-06	2.30E-05	1.25E-06	4.75E-06	9.72E-07	3.70E-06	1.63E-07	2.09E-05	1.27E-07	1.63E-05
PBT	3.86E-07	8.95E-07	4.41E-07	1.02E-06	1.52E-07	1.55E-06	1.74E-07	1.77E-06	3.27E-08	1.60E-06	2.55E-08	1.24E-06	3.50E-09	3.51E-08	2.73E-09	2.73E-08
PBEB	5.11E-08	6.86E-08	5.85E-08	7.85E-08	4.93E-08	2.30E-07	5.64E-08	2.63E-07	1.78E-08	1.16E-06	1.39E-08	9.07E-07	-	-	-	-
BTBPE	4.58E-07	2.35E-06	5.24E-07	2.69E-06	1.28E-06	5.40E-06	1.46E-06	6.18E-06	6.88E-08	1.53E-07	5.36E-08	1.19E-07	4.39E-08	3.23E-06	3.42E-08	2.52E-06
∑6NBFRs	7.76E-06	1.82E-05	8.88E-06	2.08E-05	8.02E-06	7.54E-05	9.18E-06	8.63E-05	2.18E-06	1.25E-05	1.70E-06	9.77E-06	2.89E-07	2.43E-05	2.25E-07	1.89E-05
α-HBCD	3.38E-06	1.58E-05	3.87E-06	1.80E-05	2.62E-06	2.04E-05	2.99E-06	2.33E-05	4.19E-07	4.66E-06	3.26E-07	3.63E-06	4.78E-08	4.38E-06	3.73E-08	3.41E-06
β-HBCD	1.18E-06	3.49E-06	1.34E-06	3.99E-06	3.71E-07	5.54E-06	4.25E-07	6.34E-06	1.43E-07	8.01E-07	1.11E-07	6.25E-07	9.45E-09	2.23E-06	7.36E-09	1.74E-06
γ-HBCD	1.05E-05	2.65E-05	1.21E-05	3.03E-05	2.03E-06	1.34E-04	2.32E-06	1.53E-04	3.64E-07	2.23E-06	2.84E-07	1.74E-06	4.81E-08	4.82E-07	3.75E-08	3.76E-07
∑3HBCDs	1.81E-05	3.21E-05	2.07E-05	3.67E-05	5.50E-06	1.58E-04	6.28E-06	1.81E-04	1.02E-06	6.12E-06	7.92E-07	4.77E-06	8.63E-08	6.81E-06	6.72E-08	5.31E-06

*the dash means the median concentration is not available.

Table S8. The estimated daily intake of NBFRs and HBCDs via dust dermal contact pathway (ng/kg bw/d).

Areas	DW1				DW2				WO				RAO			
Gender	Male		Female		Male		Female		Male		Female		Male		Female	
EDIs	Average	High	Average	High	Average	High	Average	High	Average	High	Average	High	Average	High	Average	High
HBBZ	8.04E-04	5.54E-03	8.39E-04	5.78E-03	-	7.53E-02	-	7.86E-02	-	8.70E-03	-	6.19E-03	-	-	-	-
EHTBB	7.07E-04	1.25E-03	7.38E-04	1.30E-03	1.19E-04	8.91E-04	1.24E-04	9.30E-04	5.90E-05	2.61E-04	4.20E-05	1.85E-04	5.08E-05	2.20E-04	3.61E-05	1.57E-04
BEHTBP	6.44E-03	2.20E-02	6.73E-03	2.30E-02	7.42E-03	2.72E-02	7.75E-03	2.84E-02	1.69E-03	6.44E-03	1.20E-03	4.58E-03	2.20E-04	2.83E-02	1.57E-04	2.01E-02
PBT	5.22E-04	1.21E-03	5.46E-04	1.27E-03	2.06E-04	2.09E-03	2.16E-04	2.19E-03	4.43E-05	2.16E-03	3.15E-05	1.54E-03	4.74E-06	4.75E-05	3.37E-06	3.38E-05
PBEB	6.93E-05	9.30E-05	7.23E-05	9.71E-05	6.69E-05	3.12E-04	6.98E-05	3.26E-04	2.41E-05	1.58E-03	1.72E-05	1.12E-03	-	-	-	-
BTBPE	6.21E-04	3.19E-03	6.48E-04	3.33E-03	1.73E-03	7.32E-03	1.81E-03	7.64E-03	9.31E-05	2.07E-04	6.63E-05	1.48E-04	5.94E-05	4.38E-03	4.23E-05	3.12E-03
∑6NBFRs	1.05E-02	2.47E-02	1.10E-02	2.58E-02	1.09E-02	1.02E-01	1.13E-02	1.07E-01	2.95E-03	1.70E-02	2.10E-03	1.21E-02	3.91E-04	3.29E-02	2.78E-04	2.34E-02
α-HBCD	4.58E-03	2.13E-02	4.79E-03	2.23E-02	3.54E-03	2.76E-02	3.70E-03	2.89E-02	5.67E-04	6.31E-03	4.04E-04	4.49E-03	6.48E-05	5.94E-03	4.61E-05	4.22E-03
β-HBCD	1.59E-03	4.73E-03	1.66E-03	4.94E-03	5.03E-04	7.51E-03	5.25E-04	7.84E-03	1.94E-04	1.09E-03	1.38E-04	7.72E-04	1.28E-05	3.03E-03	9.10E-06	2.15E-03
γ-HBCD	1.43E-02	3.58E-02	1.49E-02	3.74E-02	2.74E-03	1.81E-01	2.86E-03	1.89E-01	4.93E-04	3.02E-03	3.51E-04	2.15E-03	6.52E-05	6.53E-04	4.64E-05	4.65E-04
∑3HBCDs	2.45E-02	4.35E-02	2.56E-02	4.54E-02	7.44E-03	2.14E-01	7.77E-03	2.24E-01	1.38E-03	8.29E-03	9.79E-04	5.90E-03	1.17E-04	9.23E-03	8.32E-05	6.57E-03

*the dash means the median concentration is not available.

Table S9. The calculated hazard quotients for NBFRs and HBCDs

Target Compounds	RfD* (ng/kg bw/d)	EDI_{sum}				HQs			
		Male		Female		Male		Female	
		Average	High	Average	High	Average	High	Average	High
HBBZ	100	2.02E-03	1.39E-02	2.23E-03	1.54E-02	2.02E-05	1.39E-04	2.23E-05	1.54E-04
EHTBB	20000	1.77E-03	3.13E-03	1.96E-03	3.46E-03	8.87E-08	1.57E-07	9.79E-08	1.73E-07
BEHTBP	20000	1.62E-02	5.53E-02	1.79E-02	6.10E-02	8.09E-07	2.76E-06	8.93E-07	3.05E-06
PBT	100	1.31E-03	3.04E-03	1.45E-03	3.36E-03	1.31E-05	3.04E-05	1.45E-05	3.36E-05
PBEB	100	1.74E-04	2.33E-04	1.92E-04	2.58E-04	1.74E-06	2.33E-06	1.92E-06	2.58E-06
BTBPE	243000	1.56E-03	8.00E-03	1.72E-03	8.83E-03	6.42E-09	3.29E-08	7.08E-09	3.64E-08
∑6NBFRs		2.64E-02	6.20E-02	2.91E-02	6.84E-02	3.59E-05	1.75E-04	3.97E-05	1.93E-04
α-HBCD	200000	1.15E-02	5.36E-02	1.27E-02	5.91E-02	5.75E-08	2.68E-07	6.35E-08	2.96E-07
β-HBCD	200000	4.00E-03	1.19E-02	4.41E-03	1.31E-02	2.00E-08	5.94E-08	2.21E-08	6.56E-08
γ-HBCD	200000	3.58E-02	9.00E-02	3.96E-02	9.93E-02	1.79E-07	4.50E-07	1.98E-07	4.97E-07
∑3HBCDs		6.15E-02	1.09E-01	6.79E-02	1.21E-01	2.57E-07	7.77E-07	2.83E-07	8.58E-07

*RfD values are all from Integrated Risk Information System (IRIS) in EPA (https://www.epa.gov/ncea/iris/index.html).
EDI_{sum} means total EDI of dust ingestion, inhalation, and dermal contact pathways.

References

EPA. Integrated Risk Information System, 1991; (www.epa.gov/ncea/iris/index.html) (accessed: August15, 2008).

ALI N, HARRAD S, GOOSEY E, et al. 2011. "Novel" brominated flame retardants in Belgian and UK indoor dust: implications for human exposure. Chemosphere [J], 83: 1360-1365.

BU Q, WU D, XIA J, et al. 2019. Polybrominated diphenyl ethers and novel brominated flame retardants in indoor dust of different microenvironments in Beijing, China. Environment International [J], 122: 159-167.

CRAES 2015. The Chinese Research Academy of Environmental Sciences. Highlights of the Chinese Exposure Factors Handbook (Adults) [M]. Academic Press, ISBN: 978-0-12-803125-4.

HOANG M T T, ANH H Q, KADOKAMI K, et al. 2021. Contamination status, emission sources, and human health risk of brominated flame retardants in urban indoor dust from Hanoi, Vietnam: the replacement of legacy polybrominated diphenyl ether mixtures by alternative formulations. Environmental Science and Pollution Research [J], 28: 43885-43896.

LING S, LU C, PENG C, et al. 2021. Characteristics of legacy and novel brominated flame retardants in water and sediment surrounding two e-waste dismantling regions in Taizhou, eastern China. Science of the Total Environment [J], 794: 148744.

LING S, ZHOU S, TAN J, et al. 2022. Brominated flame retardants (BFRs) in sediment from a typical e-waste dismantling region in Southern China: Occurrence, spatial distribution, composition profiles, and ecological risks. Science of the Total Environment [J], 824: 153813.

NGUYEN L V, DIAMOND M L, VENIER M, et al. 2019. Exposure of Canadian electronic waste dismantlers to flame retardants. Environ Int [J], 129: 95-104.

TORRE A D L, NAVARRO I, SANZ P, et al. 2020. Organophosphate compounds, polybrominated diphenyl ethers and novel brominated flame retardants in European indoor house dust: Use, evidence for replacements and assessment of human exposure. Journal of Hazardous Materials [J], 382: 121009.

USEPA 2011. Exposure factors handbook [M], https://www.epa.gov/expobox/exposure-factors-handbook-2011-edition.

WANG J, MA Y, CHEN S, et al. 2010. Brominated flame retardants in house dust from e-waste recycling and urban areas in South China: implications on human exposure. Environment International [J], 36: 535-541.

WANG Y, ZHANG Y, TAN F, et al. 2020. Characteristics of halogenated flame retardants in the atmosphere of Dalian, China. Atmospheric Environment [J], 223.

WANNOMAI T, MATSUKAMI H, UCHIDA N, et al. 2020. Bioaccessibility and exposure assessment of flame retardants via dust ingestion for workers in e-waste processing workshops in northern Vietnam. Chemosphere [J], 251: 126632.

ZHANG Q, LI X, WANG Y, et al. 2021. Occurrence of novel organophosphate esters derived from organophosphite antioxidants in an e-waste dismantling area: Associations between hand wipes and dust. Environment International [J], 157: 106860.

ZHAO L, LU Y, ZHU H, et al. 2022. E-waste dismantling-related occupational and routine exposure to melamine and its derivatives: Estimating exposure via dust ingestion and hand-to-mouth contact. Environment International [J], 165: 107299.

ZHENG X, XU F, CHEN K, et al. 2015. Flame retardants and organochlorines in indoor dust from several e-waste recycling sites in South China: composition variations and implications for human exposure. Environ Int [J], 78: 1-7.
